# Lusutrombopag (Mulpleta®) treatment in a patient with thrombocytopenia complicated by cirrhosis prior to continuous epidural anesthesia during renal artery embolization: a case report

**DOI:** 10.1186/s40981-018-0217-7

**Published:** 2018-11-20

**Authors:** Tomoko Sasaguri, Naomi Hirakawa, Satoko Uemura

**Affiliations:** Pain Clinic and Palliative Care Medicine, Saga University Hospital, 5-1-1 Nabeshima, Saga, 849-8501 Japan

**Keywords:** Lusutrombopag, Epidural anesthesia, Pain management, Thrombopoietin, Thrombocytopenia, Liver cirrhosis

## Abstract

**Background:**

The oral thrombopoietin (TPO) receptor agonist lusutrombopag (Mulpleta®) was developed to improve thrombocytopenia in patients with chronic liver disease prior to elective invasive medical procedures. Mulpleta® was first approved for use in Japan in 2015 and in the USA in 2018. In the present report, we discuss a case in which pain management was performed during left renal artery embolization via continuous epidural anesthesia following oral administration of lusutrombopag. To our knowledge, this is the first report to discuss the use of lusutrombopag prior to epidural anesthesia.

**Case presentation:**

The patient was a 78-year-old woman scheduled to undergo renal artery embolization to address a 3-cm aneurysm of the left renal artery. Fourteen days prior to the scheduled embolization procedure, the urologist was asked to insert an epidural catheter for perioperative and postoperative analgesia. Type C chronic cirrhosis was observed, and platelet count was 5.6 × 10^4^/μL. Eleven days prior to embolization, oral lusutrombopag was initiated at a dosage of 3 mg/day (day 1). Oral lusutrombopag therapy was continued for 5 days, and platelet count on day 11 (i.e., the day prior to surgery) was 12.6 × 10^4^/μL. An epidural catheter was inserted on day 12, following which embolization was performed. Platelet count on day 13 was 11.0 × 10^4^/μL, and the catheter was removed on day 14. No symptoms of epidural hematoma or thrombosis were observed during the patient’s disease course.

**Conclusions:**

As lusutrombopag is a relatively safe platelet-increasing agent, we believe that this drug can serve as a potential treatment option when performing elective epidural anesthesia in patients with chronic liver disease complicated by thrombocytopenia.

## Background

In cases of chronic liver disease, multiple invasive procedures are often necessary, including endoscopic treatment for esophageal or gastric varices or hepatic cancer treatment with radiofrequency ablation. Although platelet transfusion is considered in many cases, complications such as inefficacy of transfusion, onset of unknown infection, or development of fever/allergic symptoms present clinical obstacles [1, 2]. The thrombopoietin (TPO) receptor agonist lusutrombopag, which is administered prior to invasive procedures, was developed to improve platelet count and avoid the need for transfusions in patients with chronic liver disease complicated by thrombocytopenia. In September 2015, lusutrombopag obtained manufacturing and marketing approval in Japan and became the world’s first therapeutic agent of its kind [3, 4]. In the present report, we discuss a case in which pain management was performed during left renal artery embolization via continuous epidural anesthesia following oral administration of lusutrombopag. To the best of our knowledge, this report is the first to discuss the use of lusutrombopag prior to epidural anesthesia.

## Case presentation

The patient was a 78-year-old woman (height 147 cm; weight 61 kg) with no history of thrombosis or embolism who was examined per the treatment guidelines of the urology, circulatory internal medicine, vascular surgery, and radiology departments of our institution. Abdominal CT revealed that a previously identified left renal artery aneurysm had expanded to 3 cm in size. No symptoms of renal aneurysm were observed, although the risk of aneurysmal rupture or dissection was high. The patient was determined to face high risks when she is to undergo surgery due to her advanced age and the presence of various conditions complicating her thrombocytopenia (e.g., cirrhosis). Fourteen days prior to the scheduled embolization of the left renal artery trunk, the urologist was asked to insert an epidural catheter for perioperative and postoperative analgesia. As renal artery embolization often results in intense pain, at least some degree of analgesic treatment is necessary during the perioperative period. However, as the patient’s platelet count had shifted to 5–6 × 10^4^/μL over the past several months, the risk of hemorrhaging during the epidural puncture was determined to be high. Therefore, lusutrombopag (Mulpleta®, Shionogi, Osaka, Japan) was administered prior to surgery. Continuous epidural anesthesia was to be administered if the patient’s platelet count increased to 10 × 10^4^/μL or higher on the day before embolization, while alternative analgesic approaches (e.g., intravenous administration of fentanyl (continuous and/or bolus), intravenous and/or oral administration of acetaminophen, and/or non-steroidal anti-inflammatory drugs) would be utilized if the platelet count was lower than 10 × 10^4^/μL.

The patient’s comorbidities included asymptomatic cirrhosis type C, postoperative hepatocellular carcinoma, ruptured esophageal varices, and postoperative breast cancer. Internal medications included ursodeoxycholic acid (600 mg × 3), an amino acid preparation for liver failure (12.45 g × 3), an oral nutritional supplement for liver failure (50 g), lactulose jelly (48.15 g × 3), spironolactone (75 mg × 1), amlodipine (5 mg × 1), tolvaptan (7.5 mg × 1), and rabeprazole sodium (10 mg × 1). Laboratory findings at 14 days prior to the scheduled embolization are shown in Table 1. The patient’s condition was categorized as class B under the Child-Pugh classification system. Abdominal CT revealed splenomegaly, although no evidence of thrombosis was noted.

**Table 1:** Laboratory data on day 3

Hematology	
White blood cells	2700 (/μL)
Red blood cells	393 × 10^4^ (/μL)
Hemoglobin	13.5 (g/dL)
Platelet count	5.4 × 10^4^ (/μL)
Coagulation	
PT%	65.9 (%)
PT-INR	1.22
Biochemistry	
Total protein	7.3 (g/dL)
Albumin	3.2 (mg/dL)
Total bilirubin	1.3 (mg/dL)
AST	59 (IU/L)
ALT	41 (IU/L)
LDH	239 (IU/L)
ALP	439 (IU/L)
GGT	22 (IU/L)
Blood urea nitrogen	11.6 (mg/dL)
Creatinine	0.58 (mg/dL)

*PT-INR* international normalized ratio of prothrombin time, *AST* aspartate aminotransferase, *ALT* alanine aminotransferase, *LDH* lactate dehydrogenase, *ALP* alkaline phosphatase, *GGT* gamma-glutamyl transpeptidase

**Fig. 1:**
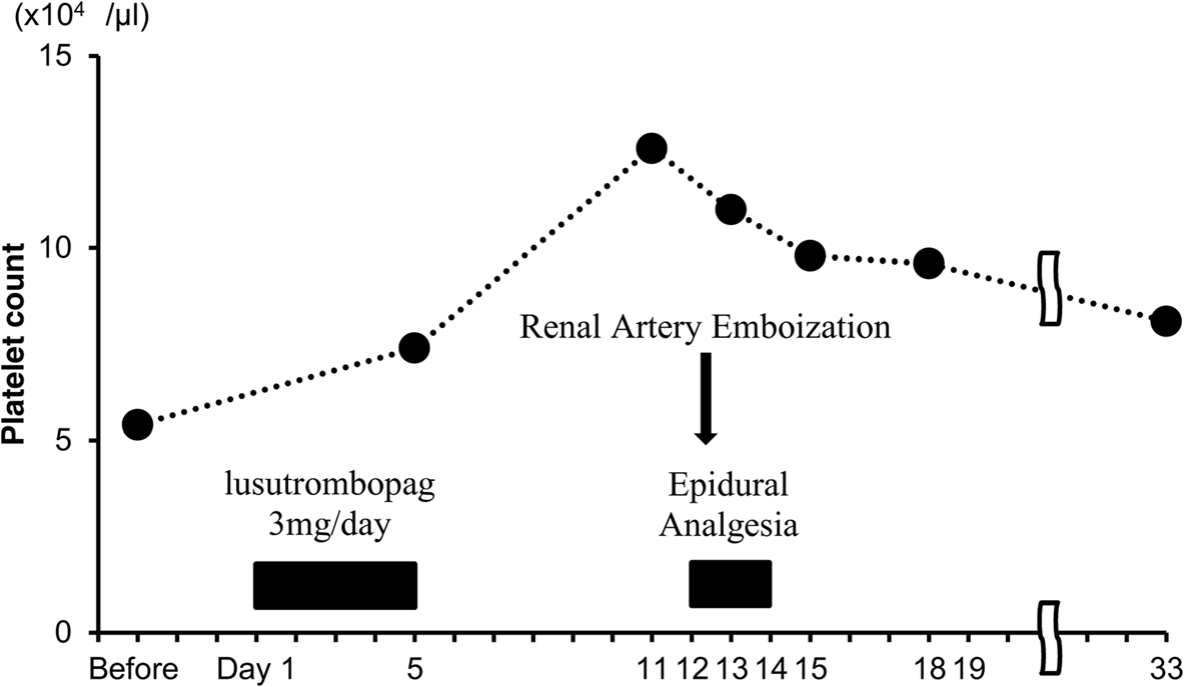
Clinical course. From day 1 to day 5, the patient was treated with 3 mg of lusutrombopag daily. Treatment was discontinued on day 5 due to an increase in platelet count (7.4 × 10^4^/μL). Platelet count on day 11 was 12.6 × 10^4^/μL, and epidural anesthesia was performed the following day. Platelet count remained at 9.0 × 10^4^/μL during hospitalization (from day 11 to day 19)

The patient’s treatment course is depicted in Fig. 1. Fourteen days prior to the scheduled embolization, she was examined by physicians of the department of hepatic internal medicine, 3 days following which she began use of oral lusutrombopag (3 mg) once daily (day 1). Although the patient’s platelet count prior to administration of lusutrombopag was 5.4 × 10^4^/μL, it increased to 7.4 × 10^4^/μL after 5 days of treatment. Then, oral lusutrombopag was discontinued to avoid the risk of thrombosis due to the increased platelet count. The patient was hospitalized on day 11. As the platelet count at the time of admission to the hospital was 12.6 × 10^4^/μL, an epidural catheter was inserted on day 12 according to the original schedule, with the following characteristics: epidural needle size, 17G; catheter type, Hakko disposable epidural catheter (Hakko, Co., Ltd., Tokyo, Japan) and nylon block copolymer radiopaque catheter; diameter, 1.0 mm; tip, round with lateral holes; level of insertion, T8–9; and insertion length, 5 cm into the epidural space. Subsequently, left renal artery embolization was performed under continuous epidural anesthesia (4 mL/h of 0.2% ropivacaine after bolus administration of 5 mL of 1% mepivacaine; analgesia before embolization from T5 to L1). Continuous epidural analgesia (4 mL/h of 0.2% ropivacaine; analgesia from T6 to T11) was also utilized following surgery, and the patient experienced no pain during the perioperative period. The platelet count on day 13 was 11.0 × 10^4^/μL, and the epidural catheter was removed on day 14. The platelet count peaked on the day before embolization and gradually decreased thereafter. The patient’s platelet counts on days 15 and 18 were 9.8 × 10^4^/μL and 9.6 × 10^4^/μL, respectively. No symptoms of epidural hematoma were observed, and the patient was released on day 19 due to her favorable postoperative course. The patient was subsequently followed-up on an outpatient basis. No symptoms of thrombosis were observed after initiating oral lusutrombopag therapy, and abdominal echo did not reveal portal thrombosis on day 33, when her platelet count was 8.1 × 10^4^/μL.

## Discussion

In the present report, we discussed a case in which continuous epidural anesthesia was performed following oral administration of lusutrombopag. Our findings suggest that lusutrombopag can be used to increase the platelet count prior to epidural anesthesia in patients with cirrhosis complicated by thrombocytopenia.

Approved in September 2015 for the “improvement of thrombocytopenia in patients with chronic liver disease scheduled to undergo an elective invasive medical procedure” [3, 4], Mulpleta® is an oral TPO receptor agonist developed and manufactured in Japan. Lusutrombopag, which has also been approved by the US Food and Drug Administration [5], selectively acts on the TPO receptor of myeloid progenitor cells to promote proliferation and differentiation of megakaryocytes, thereby increasing the platelet count [6]. While two other drugs with similar pharmacological mechanisms (eltrombopag, romiplostim) are currently marketed in Japan, these drugs are indicated for idiopathic thrombocytopenic purpura and not for the treatment of thrombocytopenia in patients with chronic liver disease. The target serum platelet count indicated by Japan’s Pharmaceuticals and Medical Devices Agency (PMDA) for considering the use of lusutrombopag treatment is < 5 × 10^4^/μL [7]. The PMDA also mentions the following: “Use Mulpleta® when a bleeding risk is high judging from clinical inspection levels such as the platelet count, clinical manifestations and the kind of the invasive procedure” [7]. This signifies that a platelet count < 5 × 10^4^/μL is only a guide for the usage of lusutrombopag. In this case, we decided to use lusutrombopag because the patient’s platelet count had shifted to 5–6 × 10^4^/μL over the past several months, and a platelet count higher than 10 × 10^4^/μL is required for epidural tubing. Careful administration of lusutrombopag is necessary in patients at risk for thrombus-related disorders such as cerebrovascular infarction and myocardial infarction and in those at risk of developing portal vein thrombosis due to hepatofugal flow of portal blood. In addition, the use of lusutrombopag is contraindicated in patients presenting with severe liver disorders (Child-Pugh class C) due to the risk of excessive increase in the serum concentrations of lusutrombopag [7]. Lusutrombopag administration is indicated for invasive procedures associated with a limited volume of bleeding and with comparatively low difficulty in achieving hemostasis. The use of lusutrombopag is not indicated in conjunction with procedures involving laparotomy, thoracotomy, cardiotomy, craniotomy, or organ resection [7].

Administration of lusutrombopag should be initiated 8–13 days prior to a scheduled invasive medical procedure, as an increase in the platelet count to 5 × 10^4^/μL or higher may be observed 9–14 days after initiating treatment [7]. Because administration is often initiated on an outpatient basis, providing medication guidance to patients is critical for preventing errors. Although 3 mg of lusutrombopag is typically administered once daily for 7 days, criteria for suspending administration have been established to avoid the onset of thrombosis due to excessive increase in the platelet count. Specifically, administration of lusutrombopag should be suspended if the platelet count increases to > 5 × 10^4^/μL and > 2 × 10^4^/μL compared to baseline [7]. Adverse reactions known at the time of lusutrombopag approval include thrombosis-related conditions (e.g., portal vein and mesenteric vein thromboses), rash, blood abnormalities, nausea, headache, fever, and others, each of which occurred in less than 2% of patients in clinical trials [7]. Although thrombosis was noted as a severe potential side effect, patients with chronic liver disease complicated by thrombocytopenia tend to develop thrombi in the portal venous system due to the activation of coagulation mechanisms and abnormalities in portal blood flow [8]. Because the risk of thrombosis/embolism formation is believed to be relatively high in patients using lusutrombopag, patients must be closely monitored for potential signs of thrombosis, even after use of the drug has been discontinued. Thrombosis development has also been reported in some patients despite the platelet counts being below the normal range [7]. Accordingly, attending physicians should monitor for evidence of thrombosis development regardless of the platelet count. In this case, we closely monitored for potential signs of thrombosis and no symptoms and signs were observed during the patient’s disease course. In addition, abdominal echo on day 33 did not reveal thrombosis or embolism. Although we did not monitor the serum value of fibrin/fibrinogen degradation products (FDPs), a biomarker of deep vein thrombosis, as no signs of thrombosis were observed, FDP monitoring could lead to early diagnosis of deep vein thrombosis. While we identified one report discussing a case in which lusutrombopag was re-administered to a patient [9, 10], at present, the safety and efficacy of lusutrombopag re-administration remain to be evaluated, and selection of an alternative treatment method is recommended [7]. However, as invasive procedures are often repeated in patients with chronic liver disease, further studies are required to determine whether re-administration of the drug is both safe and effective. Patients with cirrhosis may have complications involving thrombocytopenia and low fibrinogen concentration. The combination of lusutrombopag and fresh frozen plasma is not contraindicated at present, but there have so far been no reports of their concurrent use. It is necessary to carefully monitor for signs of the thrombosis when they are used together.

In the present case, although the patient’s platelet count prior to administration of lusutrombopag was 5.4 × 10^4^/μL, it increased to 7.4 × 10^4^/μL after 5 days of oral treatment. In addition, the platelet count peaked at 12.6 × 10^4^/μL on day 11, gradually decreasing thereafter and failing to reach 20 × 10^4^/μL or higher, which is considered the threshold for the risk of thrombus formation [11]. The patient was carefully monitored during her course for signs of complications such as thrombosis or epidural hematoma, and no clear side effects were observed.

## Conclusions

To our knowledge, the present report is the first to discuss the use of lusutrombopag prior to epidural anesthesia. Lusutrombopag is a relatively safe platelet-increasing agent, and we believe that this drug can serve as a potential treatment option when performing elective epidural anesthesia in patients with chronic liver disease complicated by thrombocytopenia.

## Abbreviations

FDP: Fibrin/fibrinogen degradation products
PMDA: Pharmaceuticals and Medical Devices Agency
TPO: Thrombopoietin
